# Social Distancing in Chronic Migraine during the COVID-19 Outbreak: Results from a Multicenter Observational Study

**DOI:** 10.3390/nu13041361

**Published:** 2021-04-19

**Authors:** Vincenzo Di Stefano, Raffaele Ornello, Andrea Gagliardo, Angelo Torrente, Elisa Illuminato, Valeria Caponnetto, Ilaria Frattale, Raffaella Golini, Chiara Di Felice, Fabiola Graziano, Maria Caccamo, Davide Ventimiglia, Salvatore Iacono, Gabriella Matarazzo, Francesco Armetta, Giuseppe Battaglia, Alberto Firenze, Simona Sacco, Filippo Brighina

**Affiliations:** 1 Department of Biomedicine, Neuroscience and Advanced Diagnostic (BIND), University of Palermo, 90127 Palermo, Italy; andrigl@gmail.com (A.G.); angelotorrente92@gmail.com (A.T.); fabiolagraz93@gmail.com (F.G.); maria.caccamo91@gmail.com (M.C.); davideventimiglia211@gmail.com (D.V.); Salvo.iak@gmail.com (S.I.); filippo.brighina@unipa.it (F.B.); 2 Department of Applied Clinical Sciences and Biotechnology, University of L’Aquila, 67100 L’Aquila, Italy; raffaele.ornello@gmail.com (R.O.); caponnettovaleria@gmail.com (V.C.); ilariafrattale@libero.it (I.F.); gabriellamatarazzo95@gmail.com (G.M.); simona.sacco@univaq.it (S.S.); 3 Department of Health Promotion, Mother and Child Care, Internal Medicine and Medical Specialties “G. D’Alessandro”, University of Palermo, 90127 Palermo, Italy; ellyna182@gmail.com (E.I.); francesco.armetta03@gmail.com (F.A.); alberto.firenze@unipa.it (A.F.); 4 Department of Internal Medicine, Public Health, Life and Environmental Sciences, University of L’Aquila, 67100 L’Aquila, Italy; golini87@gmail.com (R.G.); chiaradi.felice@yahoo.com (C.D.F.); 5 Department of Psychology, Educational Science and Human Movement, University of Palermo, 90127 Palermo, Italy; giuseppe.battaglia@unipa.it

**Keywords:** COVID-19, migraine, quarantine, physical activity, nutrition, sleep disorder

## Abstract

Background: The restrictions taken to control the rapid spread of COVID-19 resulted in a sudden, unprecedented change in people’s lifestyle, leading to negative consequences on general health. This study aimed to estimate the impact of such changes on migraine severity during 2020 March–May lockdown. Methods: Patients affected by migraine with or without aura, diagnosed by expert physicians, completed a detailed interview comprehensive of: assessment of migraine characteristics; measure of physical activity (PA) levels; measure of the intake frequency of main Italian foods; the Insomnia Severity Index (ISI) questionnaire investigating sleep disorders. Results: We included 261 patients with a mean age of 44.5 ± 12.3 years. During social distancing, 72 patients (28%) reported a headache worsening, 86 (33%) an improvement, and 103 (39%) a stable headache frequency. A significant decrease of the PA levels during COVID-19 quarantine in the whole study sample was observed (median total metabolic equivalent task (METs) decreased from 1170 to 510; *p* < 0.001). Additionally, a significant difference was reported on median ISI scores (from 7 to 8; *p* < 0.001), which were increased in patients who presented a stable or worsening headache. Conclusions: Our study confirmed that the restrictions taken during the pandemic have affected the practice of PA levels and sleep quality in migraine. Hence, PA and sleep quality should be assessed to find strategies for an improvement in quality of life.

## 1. Introduction

COVID-19 was declared a public health emergency in many countries [1,2]. Hence, restrictive measures and social distancing were imposed by the governments, influencing many aspects of everyday life, from the practice of physical activity (PA) to eating habits and sleep compliance [3,4]. Indeed, many people were asked to live in home-confinement for several months, becoming inactive and sedentary [4,5,6,7]. Several studies recently appeared warning about the possible negative consequences of quarantine due to changes in nutrition, sleeping habits, and psychological distress [1,7,8]. Moreover, the sudden change in lifestyle habits might have had negative effects in patients affected by migraine, as, apart from well-established genetic [9], external factors have a known impact on migraine susceptibility [10,11,12,13]. Indoor and outdoor environmental factors, including barometric pressure changes, bright sunlight, flickering lights, air quality, and odors, are usually reported as migraine triggers [14]. Additionally, conditions such as shift working, with a consequent disruption of the sleep–wake cycle and increased levels of stress, can contribute to an aggravation of migraine episodes [15].

Recent studies recently pointed out the reduction of PA levels in the general population during the COVID-19 pandemic and its consequences in healthy subjects or in athletes [8,16,17], however, few studies have examined the consequences of quarantine in patients affected by migraine [11,18]. A recent survey exploring the effects of social distancing on subjects with migraine reported that they had fewer migraine attacks and lesser pain [11]. Unfortunately, these studies are not focused on physical activity, eating habits, and sleep.

It is a well-known fact that inactivity and sedentarism can lead to early specific alterations in the skeletal muscle [19,20,21,22]. In particular, high intensity PA is reported as a precipitant factor for migraine [23], while regular and moderate PA show a protective role in such patients [24,25]. Thus, Varkey et al. (2008) showed a strong linear trend of higher prevalence of low PA with increasing headache frequency [26]. Moreover, individuals with migraine and other headaches have been shown to be less physically active than those without headaches. Specific foods, ingredients within foods, and beverages have been identified as possible migraine triggers when introduced or eliminated from patients’ daily diet [27]. Several authors discussed and investigated possible mechanisms of action for these triggers and, consequently, useful dietary regimens to prevent migraine attacks [27,28,29,30]. In this framework, it is fundamental for migraine patients to identify dietary triggers to appropriately manage them [29], especially in the emergency setting [30,31].

Furthermore, sleep is a vulnerable stage of the circadian rhythm, as it is influenced by biological, environmental (e.g., exposure to natural light), and behavioral factors, and its vulnerability is amplified in the emergency contexts [32]. Hence, the different circadian habits associated with stress, anxiety, or depression, and reduced PA may accentuate patients’ sleep disorders and, consequently, their daily functioning with a potential increase in migraine attacks frequency [33,34,35].

In our opinion, the pandemic, through the study of abruptly changing people’s lifestyle, may offer the unique opportunity to confirm or discover the role of different environmental factors in the migraine pathogenesis. Notwithstanding the relevant impact of the COVID-19 pandemic on general health, scientific task forces have provided recommendations for the care of migraine [36,37]. However, very little has been discussed about the fundamental role of PA and eating habits during the lockdown. It has been reported that isolation at home can lead to poor nutrition, low sleep quality, reduced PA levels, and sedentary habits with different negative consequences [6,16,38]. Therefore, it is important to highlight how these unwanted consequences of quarantine may lead clinicians to develop and provide practical and useful recommendations.

In the present study, we aimed to evaluate the impact of social distancing on migraine frequency and severity; moreover, we aimed to explore the impact of COVID-19 lockdown on PA, eating habits, and sleep quality in patients affected by migraine and to verify if the lifestyle modifications detected could have contributed to any modification of migraine condition.

## 2. Materials and Methods

### 2.1. Study Design and Participants

An observational survey was conducted through a single detailed interview. To be eligible for the study, patients had to be diagnosed with migraine with or without aura according to the International Classification of Headache Disorders, 3rd Edition [39], by an expert neurologist. We included all consecutive patients aged ≥18 years referring to the study centers of Palermo and Avezzano (southern and central Italy) for a follow-up visit. Patients were instructed to keep an updated diary noting the characteristics of migraine (frequency, severity, and associated features); also, patients had to be under constant preventive medication (or no medication) during the assessment period. Hence, patients changing their preventive medication between March and June 2020 were excluded from the study. All data were collected in a single interview after the social distancing in the period between 1 May 2020 and 1 July 2020 (collecting data for both before and during lockdown conditions) [1,2]. The “social distancing period” was considered from 9 March 2020 to 3 May 2020 after the national ordinance of lockdown was imposed by the Italian government. Before being admitted to the research, all participants signed an informed consent. The Ethical Board of the University of Palermo approved the study in conformity with the Declaration of Helsinki principles.

### 2.2. Data Collection

The data were collected through a detailed questionnaire consisting of four parts directly administered to the participants. The first part regarded migraine characteristics (triggers, frequency and intensity of headache, treatment) based on the patient’s diary of migraine. The remaining three parts consisted of questionnaires on PA, eating habits, and sleep, which were collected regarding the period before and during social distancing. The adapted version of the International Physical Activity Questionnaire Short-Form (IPAQ-SF) allowed us to assess, at the same time, the levels of PA both before and during the last 7 days of COVID-19 quarantine [4,17,40]. The levels of PA were measured as energy expenditure (metabolic equivalent task (MET)–minutes/week) [40]. The adapted version of the IPAQ-SF comprised 31 questions assessing frequencies and durations of each PA intensity, e.g., sitting, walking, moderate-intensity physical activities, and vigorous-intensity physical activities. In particular, the questionnaire included questions about: demographic and anthropometric data; PA before quarantine; type of work done during quarantine; type of house lived in during quarantine; vigorous-intensity PA before and during quarantine; moderate-intensity PA before and during quarantine; walking activities before and during quarantine; sitting activities before and during quarantine; information concerning the practice of PA in home setting during quarantine [17]. An adapted version of the Food Frequency Questionnaire (FFQ) (Food Frequency Questionnaire at a glance, https://dietassessmentprimer.cancer.gov/profiles/questionnaire/, accessed on 26 February 2021) was administered to each patient. This questionnaire consisted of several items regarding the frequency of intake of 34 different kinds of foods. Finally, the Insomnia Severity Index (ISI) questionnaire regarding the quality of sleep was administered [41].

### 2.3. Scoring Protocol

Migraine patients were classified as “worsened” if the monthly frequency of headache resulted in increased during social distancing compared to before quarantine; in the opposite case, patients were considered “improved”. Finally, migraineurs were defined “stable” if the frequency of migraine attacks did not change between before and during social distancing. As for the IPAQ-SF adapted version, we considered the total PA level for both before and during quarantine. The weekly PA levels of both considered parameters were calculated as energy expenditure in MET–minutes/week (MET–min/week) [42]. We used the corresponding metabolic equivalent task assigned to each type of PA (e.g., 3.3, 4.0, and 8.0 for walking, moderate-intensity physical activities, and vigorous-intensity physical activities, respectively) to estimate the weekly level of energy expenditure. Afterwards, we computed the sum of energy expenditure of walking, moderate-intensity physical activities, and vigorous-intensity physical activities in MET–min/week for the “total PA” level [17,43]. Hence, we multiplied the corresponding MET basal level for each type of PA per minutes of practice during the week in order to calculate the MET weekly level [17,43]. The distribution of the PA level difference between before and during quarantine was calculated for each different PA intensity and for the total PA level (i.e., the sum of walking, moderate-intensity physical activities, and vigorous-intensity physical activities) in all subjects. Furthermore, we analyzed both parameters in relation to gender, age, body mass index (BMI), headache frequency, severity, and clinical variables. The food intake questionnaire presented a score indicating the monthly frequency of consumption for each item. The ISI score was calculated by simple summation of single scores from each item as for its scoring protocol [41].

### 2.4. Statistical Analysis

We adopted absolute numbers and percentages to report categorical variables and medians with interquartile range (IQR) to report distributed continuous variables. We compared patients’ characteristics before social distancing among the three groups of patients reporting increased, decreased, or unchanged headache frequency during social distancing. We compared categorical variables using the Chi-square test and continuous variables through the Mann–Whitney U test. We also performed paired analyses to assess the change in migraine characteristics, physical activity, dietary habits, and sleep quality before and during social distancing. Those paired analyses were performed with chi-square test or Wilcoxon signed rank test as appropriate. Lastly, we assessed correlations between the change in headache frequency and in lifestyle variables during social distancing by means of the Spearman’s correlation coefficient. We performed all tests using SPSS Statistic (v26, https://www.ibm.com/docs/en/spss-statistics/26.0.0, accessed on 18 April 2021) and established the level of significance at <0.05.

## 3. Results

### 3.1. Study Population

We recruited 289 patients affected by migraine in the study period. Among them, 261 agreed and completed the whole questionnaire and only 28 dropped. Among patients included, 186 (71.3%) had chronic migraine, 227 patients (87.0%) were female, and the mean age of patients was 44.5 ± 12.3 years. Depending on headache frequency during social distancing, 72 patients (27.6%) reported an increasing headache frequency, 86 (33.0%) had a reduction, and 103 (39.5%) presented a stable frequency. Patients were assigned to these three groups, which presented similar characteristics with not significant differences in age, male/female ratio, and number (Table 1). Box plots of changes in median METs are shown in Figure S1, while Table S1 displays changes in physical activity according to headache frequency change during social distancing (Supplementary Materials).

### 3.2. Physical Activity Levels and “Smart-Working”

**Figure 1:**
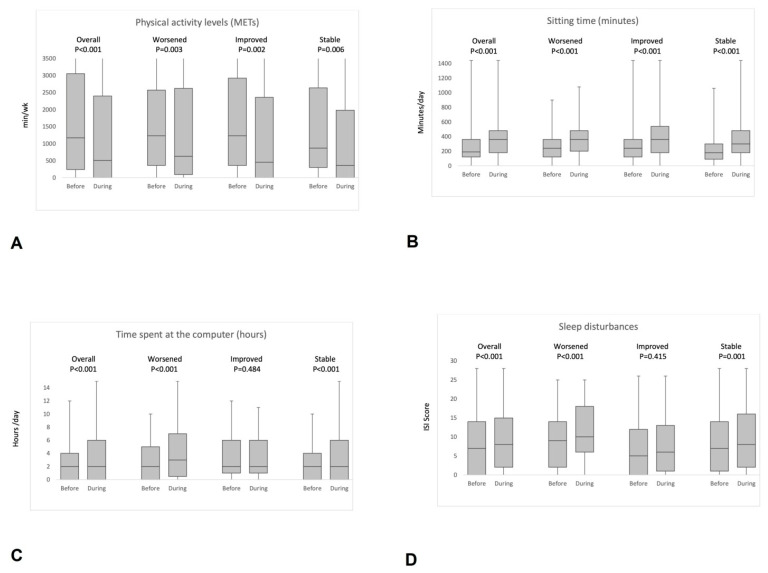
Box plots of changes in median metabolic equivalent task (METs) (**A**), sitting time (**B**), computer time (**C**), and median Insomnia Severity Index (ISI) (**D**) according to migraine frequency variation during social distancing.

In the overall group, median total METs significantly decreased during social distancing from 1170 to 510 (*p* < 0.001), driven by the decrease in median walking METs from 360 to 0 (*p* < 0.001). The decrease in total and walking METs (W-MET) was significant in all subgroups (Figure 1). A decrease in METs for vigorous activity was found in the overall group (*p* = 0.005) and in patients reporting stable headache frequency (*p* = 0.013). Sitting time correspondingly increased in the overall group and in each subgroup (*p* < 0.001 for all comparisons). Time spent at the computer significantly increased in the overall group (*p* < 0.001), in patients reporting headache worsening (*p* < 0.001), and in those reporting stable headache frequency (*p* < 0.001), while it did not significantly increase in patients reporting an improvement in their headache (*p* = 0.484). Finally, the patients’ occupational status was not associated with the course of migraine during social distancing (Table 1).

### 3.3. Eating Habits and Lifestyle

The changes in mean food portions eaten by the patients during social distancing are reported in detail in Figure S1, while Table S2 shows changes in mean monthly portions of food according to headache frequency change during social distancing (Supplementary Materials). Patients with migraine reported a significant increase in the consumption of carbohydrate-rich foods such as bread, pasta, potatoes, and cereal (*p* < 0.001), eggs (*p* = 0.025), sweets (*p* < 0.001), alcohol-free beverages (*p* = 0.003), and vegetables (*p* = 0.003). A significantly increased consumption of carbohydrate-rich foods and sweets was found across all subgroups. In the overall group and in the subgroup of patients reporting stable headache, a positive correlation was found between an increased consumption of dairy or fruit and increased walking METs. The median monthly number of cigarettes smoked marginally decreased from 210 to 190 (*p* = 0.045); however, the decrease was not significant in the three subgroups (*p* = 0.678, *p* = 0.208, and *p* = 0.130, respectively).

### 3.4. Sleep Quality

Social distancing was associated with poor sleep quality, as shown by increased ISI scores (Table 2). When considering patient subgroups, the increase in ISI scores was found in those reporting headaches worsening and stable headache, while patients reporting an improvement in their headache only reported slightly increased scores of initial and intermediate insomnia (ISI1a and ISI1b items; Table 2). The Spearman’s correlation analyses (Supplementary Materials) showed that an increase in ISI score was associated with an overall increase in headache frequency and intensity during social distancing, driven by the positive correlation found only in the subgroup of patients reporting a migraine worsening; an increase of ISI scores also correlated with a decrease of walking METs. In our migraine patients, the median total ISI score increased during social distancing (*p* < 0.001) with particular regard to the difficulty in falling asleep score (ISI1a) and in staying asleep (ISI1b), which were significantly increased in all subgroups. Patients who worsened had significant problems waking up too early (ISI1c: *p* < 0.001), dissatisfaction with their sleep pattern (ISI2: *p* = 0.002), and interference of the sleep problem with their daily functioning (ISI5: *p* = 0.004); this alteration pattern was not shown in the improved patients (*p* = 0.351), but a slight increase of the ISI1c (*p* = 0.010), ISI5 (*p* = 0.001), and total ISI score (*p* = 0.001) was also documented in the stable patients.

Finally, Table S3 (Supplementary Materials) shows the correlation between change in headache frequency and change in lifestyle habit scores during social distancing.

## 4. Discussion

In this study, we explored the impact of the COVID-19 quarantine on lifestyle of patients affected by migraine, and we investigated if these changes in lifestyle could have provoked a relevant clinical modification.

Previous studies have shown the effects of social distancing on PA in healthy subjects [17,24]. As in the general population, in our study, a significant reduction in PA levels during the pandemic was demonstrated in subjects affected by migraine (Table 2). This was in agreement with a recent study that reported a significant reduction of total weekly energy expenditure before the COVID-19 quarantine in healthy subjects [17]. Busch and Gaul (2008) summarized that regular and structured PA is often recommended in migraine treatment. In particular, aerobic endurance training appears to have beneficial effects on both frequency and intensity of migraine as well as on the time of the attacks and on patient well-being [24]. Of interest, social distancing has affected the practice of PA also in patients affected by several kinds of neuromuscular diseases, and low PA levels have been associated with a relevant impact on the quality of life [4,31,44]. Additionally, the reduction of PA was associated with alterations of sleep, as demonstrated by the ISI scores.

During COVID-19 lockdown, dietary habits of migraineurs were less healthy, since patients reported a significant increase in the consumption of palatable foods, highlighting their frustration for the current situation [31,45]. This changing was reported both in patients who experienced a headache worsening and in patients who experienced a headache improvement or stability. Probably, migraine patients were not very sensitive to dietary triggers during lockdown, and they were probably more influenced by other factors, such as sleep quality. However, a positive correlation between an increased consumption of healthy foods and walking METs was described in patients who reported a stable headache frequency. These results might suggest that healthier dietary and lifestyle habits may have contributed to avoiding migraine worsening during the social distancing.

Moreover, during quarantine, all patients complained of a worsening sleep quality according to existing evidence in the general population during the COVID-19 outbreak [33,34,35,46]; particularly, patients with migraine worsening during quarantine showed a more pronounced worsening of sleep with difficulty in falling asleep, early awakenings, dissatisfaction of sleep pattern, and interference with daily daytime activities. As sleep complaints are generally reported in metanalyses and studies of migraine triggers [47,48,49,50], we hypothesized that both the anxious state and the reduced PA might have induced difficulty in falling asleep due to a state of hyper-arousal which, in turn, might have contributed to sleep fragmentation, early awakenings, and reduced deep sleep. Fragmented sleep is usually perceived as non-restorative and negatively affects daytime performance; moreover, reduced sleep efficiency and increased wake after sleep onset was recently identified as a trigger for migraine [51].

A possible pathogenetic role of sleep disorders in migraine has been hypothesized [52]. For instance, sleep fragmentation may decrease pain thresholds [53]; furthermore, monoaminergic dysregulation in sleep disorders might increase the risk of migraine attacks. Indeed, serotonin release regulates pain, mood, and sleep, promoting wakefulness and inhibiting rapid-eye-movement (REM) sleep; in migraines, a low serotoninergic tone has been described [54,55] with an early alterations and cortical spreading depression during acute attacks reported in animal models [56]. Additionally, prodromal insomnia with an increased number of awakenings is reported in the night preceding a migraine attack. Dopamine can also modulate the trigemino-vascular system, and it also favors the arousal system of sleep [57]. Hence, the identification and the treatment of both sleep disorders and migraine might reduce the risk of chronicization [58].

In our cohort of migraineurs, the worsening of sleep was connected with the reduction of PA (W-MET); of interest, this correlation was already reported in the general population, imputed to multiple physiological and psychological factors. Indeed, a sufficient sleep and a regular PA are recommended for general public health. The American College of Sports Medicine and the American Heart Association advise regular moderate-intensity aerobic PA or vigorous-intensity aerobic activity for good health [59,60]. Acute and chronic PA promote deep sleep, increasing the EEG delta power of not rapid-eye-movement (NREM) sleep and decreasing fragmentation and wake after sleep onset both in humans [61,62] and in animal models [63]. Sleep could be also promoted by a decrease of temperature in the brain. Since PA induces changes in the body temperature during exercise and recovery [64], it could be hypothesized that PA immediately before sleep could impair sleep; conversely, in the recovery phase, the heat dissipation by a peripheral vasodilatation could promote sleep. Indeed, it has been reported that, during sleep, reduced sympathetic and increased parasympathetic tone and the down-regulation of the hypothalamo–pituitary axis reduce heart rate and blood pressure by reduced levels of cortisol, epinephrine, and norepinephrine [65]. With a similar effect, regular PA can improve vagal tone and heart rate variability, thus improving sleep [66]. This mechanism also explains how PA and the increased vagal tone could improve insomnia and the associated sympathetic mediated hyper-arousal state and how a behavioral treatment could reduce the risk of the insomnia associated depression. Allodynia and pericranial tenderness have been associated with sleep features in chronic headache patients, especially in the presence of fibromyalgia and migraine [67]. For all these reasons, we recommend exercise training and treatment for sleep disorders, particularly in low pain threshold and frequent mood disordered migraines.

Finally, social distancing has surprisingly shown a global positive effect on migraine patients. This result is in line with several recent studies [12,68,69,70,71,72]. Our data show that the patients’ occupational status was not associated with the course of migraine during social distancing (Table 1). This lack of association between occupation and change in the migraine course is in line with previous studies [12,69]. A previous study found that migraine worsening during the COVID-19 pandemic was associated with altered employment status, including remote working and high work pressure, higher levels of stress, anxiety, and worries, insomnia, wearing a face mask at work, and being confined [11]. Our survey cannot provide insight over possible changes in the work environment, except from remote working or “smart working”, which, however, did not show any association with migraine worsening (Table 1).

We found an association between a decrease in sleep quality and migraine worsening, in line with previous literature [12,71]. Our finding suggests that decreased sleep quality contributed to migraine worsening during social distancing. On the other hand, a Dutch study found an overall migraine improvement during social distancing in a population of patients with improved sleep quality [72]. That finding reinforces the suggestion that sleep quality is inversely related to migraine frequency.

## 5. Limitations

This is a survey exploring the effect of COVID-19 quarantine in PA levels, nutrition, and sleep of migraine patients. Our results come from the comparison of two conditions, “pre” and “during” quarantine; however, the evaluation of the two conditions comes from the same interview, and this may have biased the collection of data. However, we speculate that the internal consistency of the respondents led them to report the same bias both for the questions covering before and during the COVID-19 quarantine. Additionally, the majority of patients enrolled presented chronic migraine; this could be explained by an inclusion bias because the survey was proposed in two large-volume headache centers to which more severe patients are referred by common neurologists. Hence, further studies are needed to clarify whether differences in PA levels could affect other domains of health and life in migraine. We did not collect data on anxiety, work, children, house, gardens, etc., as in the full version of the IPAQ, but we used an adapted version focusing on physical activity levels. Sleep data did not include standardized questionnaires or recordings in order to classify specific sleep disorders according to current the International Classification of Sleep Disorders [73], but were limited to the Insomnia Severity Index score in order to screen subjective data on sleep quality before and during lockdown. However, we did not perform a polysomnographic or an actigraphic study of the sleep–wake pattern, and fragmented sleep was not objectively documented in our cohort but just subjectively noted by means of the ISI questionnaire. Finally, we did not report data on follow-up and migraine disability scale (MIDAS) for migraine. Future studies monitoring PA levels, nutrition, and sleep for several months after lockdown could offer new and interesting clues to the effects of lockdown and the mechanisms of recovery from sedentary behaviors.

## 6. Conclusions

This study highlights the negative impact of the measures adopted to contain COVID-19 spread on PA, eating habits, and sleep in migraine patients. An overall headache improvement was reported in 33% of patients, and only 28% presented worsening during the social distancing. Surprisingly, migraine patients were not very sensitive to dietary triggers during lockdown, and they were probably more influenced by other factors. Our study confirmed that the lockdown affected the practice of PA levels and sleep quality among patients with migraine. A significant reduction in PA was reported in patients with migraine, especially regarding walking activities. Moreover, the reduction of PA levels was associated with a worsening sleep. Therefore, since outdoor activities were limited due to the quarantine, it is essential to maintain an active lifestyle by performing exercise in a home-based setting for healthy subjects as well as for patients affected by migraine. Future studies are needed to better explore the connection between lifestyle and migraine.

## Figures and Tables

**Table 1:** Demographic and headache characteristics of the study sample.

	Overall (n = 261)	Worsened (n = 72)	Improved (n = 86)	Stable (n = 103)	*p* Value
Female, n (%)	227 (87.0)	63 (87.5)	75 (87.2)	89 (86.4)	0.975
Age, median (IQR)	45 (34.5–53)	45 (33.5–52)	45 (34–52)	46 (35–56)	0.689
BMI, median (IQR)	24 (21–26.5)	24 (21–26)	24 (21–26)	24 (21–27)	0.887
Occupation					0.632
Employed	162 (62.1)	4 (55.6)	57 (66.3)	65 (63.1)	
Unemployed	71 (27.2)	23 (31.9)	22 (25.6)	26 (25.2)	
Student	22 (8.4)	8 (11.1)	6 (7.0)	8 (7.8)	
Retired	6 (2.3)	1 (1.4)	1 (1.2)	4 (3.9)	
Works on video terminals, n (%)	92 (35.2)	25 (34.7)	33 (38.4)	34 (33.0)	0.815
Smart working during social distancing, n (%)	79 (30.2)	24 (33.3)	26 (30.2)	29 (28.2)	0.755
Chronic migraine, n (%)	186 (71.3)	50 (69.4)	68 (79.1)	68 (66.0)	0.131
Aura, n (%)	69 (26.4)	18 (25.0)	27 (31.4)	24 (23.3)	0.458
Triggers, n (%)	174 (66.7)	52 (67.8)	60 (69.8)	62 (60.2)	0.904
Food	4 (1.5)	1 (1.4)	1 (1.4)	2 (1.9)	
Climate	22 (8.4)	7 (9.7)	7 (8.1)	8 (7.8)	
Hormonal	23 (8.8)	6 (8.3)	10 (11.6)	7 (6.8)	
Psychic	58 (22.2)	17 (23.6)	19 (22.1)	22 (21.4)	
Multiple	67 (25.7)	21 (29.2)	23 (26.7)	23 (22.3)	
Monthly headache days, median (IQR)					
Before social distancing	10 (5–16)	8 (4–12)	13 (7–18)	10 (5–20)	<0.001
During social distancing	8 (4–16)	12 (8–20)	6 (2–9)	10 (5–20)	<0.001
Difference (*p* value)	0.112	<0.001	<0.001	0.999	
Headache intensity, median (IQR)					
Before social distancing	7 (6–8)	7 (5–8)	8 (6–8)	7 (6–8)	<0.001
After social distancing	7 (6–8)	8 (6–8)	6 (4–7)	8 (7–8)	<0.001
Difference (*p* value)	0.638	<0.001	<0.001	0.062	
Preventive treatment, n (%)	145 (55.6)	42 (58.3)	43 (50.0)	60 (58.3)	0.449
Stopped during social distancing	24 (16.6)	4 (9.5)	7 (16.3)	13 (21.7)	0.267

BMI: body mass index; IQR: interquartile range.

**Table 2:** Changes in mean Insomnia Severity Index (ISI) items and total score according to headache frequency change during social distancing. Numbers in parentheses indicate *p* values. Increasing scores mean worse sleep.

		Overall (n = 261)	Worsened (n = 72)	Improved (n = 86)	Stable (n = 103)
ISI1a	Before	0 (0–2)	1 (0–2)	0 (0–2)	1 (0–2)
(difficulty falling asleep)	During	1 (0–3)	1 (0–3)	0 (0–3)	1 (0–3)
	*p* value	<0.001	<0.001	0.011	0.001
ISI1b	Before	0 (0–2)	1 (0–2)	0 (0–2)	0 (0–2)
(difficulty staying asleep)	During	1 (0–3)	2 (0–3)	1 (0–2)	1 (0–3)
	*p* value	<0.001	0.001	0.046	0.006
ISI1c	Before	0 (0–2)	0 (0–2)	0 (0–2)	0 (0–2)
(problems waking up too early)	During	0 (0–2)	1 (0–3)	0 (0–2)	0 (0–2)
	*p* value	<0.001	<0.001	0.506	0.010
ISI2	Before	2 (0–3)	2 (1–2)	2 (0–3)	2 (0–3)
(satisfied/dissatisfied with sleep pattern)	During	2 (0–3)	2 (1–3)	1 (0–3)	2 (0–3)
	*p* value	0.020	0.002	0.693	0.469
ISI3	Before	1 (0–3)	1 (0–2)	0 (0–2)	1 (0–3)
(interference with daily functioning)	During	1 (0–3)	1 (0–3)	0 (0–2)	1 (0–3)
	*p* value	0.214	0.079	0.799	0.207
ISI4	Before	0 (0–2)	1 (0–2)	0 (0–2)	0 (0–2)
(noticeability of impairment)	During	0 (0–2)	1 (0–2)	0 (0–1)	0 (0–2)
	*p* value	0.543	0.166	0.469	0.507
ISI5	Before	0 (0–2)	1 (0–2)	0 (0–2)	1 (0–2)
(worried/distressed about sleep problem)	During	1 (0–2)	2 (0–3)	0 (0–2)	1 (0–3)
	*p* value	<0.001	0.004	0.463	0.001
Total score	Before	7 (0–14)	9 (2–14)	5 (0–12)	7 (1–14)
	During	8 (2–15)	10 (6–18)	6 (1–13)	8 (2–16)
	*p* value	<0.001	<0.001	0.415	0.001

## Data Availability

The datasets generated for this study are available on request to the corresponding author.

## Supplementary Materials

The following are available online at https://www.mdpi.com/article/10.3390/nu13041361/s1, Figure S1: Box plots of changes in median METs (A), median monthly portions of carbohydrate-rich foods (B) and sweets, Table S1: Changes in physical activity according to headache frequency change during social distancing, Table S2: Changes in mean monthly portions of food according to headache frequency change during social distancing, Table S3: Correlation between change in headache frequency and change in lifestyle habit scores during social distancing.
