# Supplementary material for: Social Distancing in Chronic Migraine during the COVID-19 Outbreak: Results from a Multicenter Observational Study
Source: Nutrients. 2021 Apr 19;13(4):1361. doi: 10.3390/nu13041361 (PMC8074143; doi:10.3390/nu13041361)
Supplement: Supplementary file 1 [file nutrients-13-01361-s001.zip › nutrients-1144347-supplementary.pdf]

Supplementary material

Supplementary Table S1. Changes in physical activity (METs-min/wk) according to headache frequency change during social distancing.

	Overall (n=261)	Worsened (n=72)	Improved (n=86)	Stable (n=103)
MET – Vigorous activity				
Before	0 (0-0)	0 (0-480)	0 (0-0)	0 (0-0)
During	0 (0-0)	0 (0-0)	0 (0-0)	0 (0-0)
P value	**0.005**	0.322	0.232	**0.013**
MET – Moderate activity				
Before	200 (0-840)	120 (0-880)	480 (0-960)	0 (0-640)
During	0 (0-900)	0 (0-720)	320 (0-1200)	0 (0-720)
P value	0.467	0.856	0.878	0.214
MET – Walking				
Before	360 (45-840)	270 (0-788)	540 (90-945)	300 (90-630)
During	0 (0-210)	0 (0-315)	0 (0-196)	0 (0-180)
P value	**<0.001**	**<0.001**	**<0.001**	**<0.001**
MET – Total				
Before	1170 (240-3055)	1230 (360-2575)	1230 (360-2925)	870 (300-2640)
During	510 (0-2400)	630 (90-2625)	450 (0-2363)	360 (0-1980)
P value	**<0.001**	**0.003**	**0.002**	**0.006**
Sitting time (minutes)				
Before	190 (120-360)	240 (120-360)	240 (120-360)	180 (90-300)
During	360 (180-480)	360 (200-480)	360 (180-540)	300 (180-480)
P value	**<0.001**	**<0.001**	**<0.001**	**<0.001**
Computer time (hours)				
Before	2 (0-4)	2 (0-5)	2 (1-6)	2 (0-4)
During	2 (0-6)	3 (0.5-7)	2 (1-6)	2 (0-6)
P value	**<0.001**	**<0.001**	0.484	**<0.001**

Supplementary Table S2. Changes in mean monthly portions of food according to headache frequency change during social distancing. Numbers in parentheses indicate P values.

		Overall (n=261)	Worsened (n=72)	Improved (n=86)	Stable (n=103)
Protein-rich	Before	16 (11-20)	15 (10-20)	16 (12-23)	15 (11-20)
	During	16 (10-20)	15 (9-20)	16 (11-24)	15 (10-20)
	P value	0.071	0.079	0.576	0.389
Carbohydrate-rich	Before	75 (56-98)	75 (55-97)	81 (62-98)	72 (55-96)
	During	78 (59-101)	77 (59-101)	88 (64-102)	75 (56-98)
	P value	**<0.001**	**<0.001**	**0.002**	**0.002**
Dairies	Before	16 (6-34)	16 (6-35)	20 (8-38)	15 (6-30)
	During	16 (8-20)	19 (10-31)	19 (9-31)	15 (4-30)
	P value	0.117	0.416	0.119	0.056
Eggs	Before	4 (4-8)	4 (4-8)	4 (4-8)	4 (3-7)
	During	4 (4-8)	4 (4-8)	4 (4-8)	4 (4-8)
	P value	**0.025**	0.482	**0.044**	0.862
Sweets/cakes/chocolate	Before	8 (2-15)	10 (4-20)	8 (4-16)	4 (1-12)
	During	12 (4-22)	14 (4-26)	14 (6-26)	8 (2-16)
	P value	**<0.001**	**0.003**	**<0.001**	**<0.001**
Sauces	Before	0 (0-4)	2 (0-4)	0 (0-4)	0 (0-4)
	During	0 (0-4)	0 (0-4)	0 (0-4)	0 (0-2)
	P value	0.159	0.420	0.104	0.579
Tea/coffee	Before	50 (30-60)	32 (30-60)	30 (30-50)	30 (30-60)
	During	30 (30-58)	35 (30-60)	30 (30-45)	30 (30-60)
	P value	0.229	0.677	0.261	0.844
Alcohol-free beverages	Before	1 (0-8)	2 (0-10)	2 (0-8)	0 (0-6)
	During	0 (0-10)	1 (0-10)	2 (0-12)	0 (0-8)
	P value	**0.003**	0.075	0.059	0.190

Alcoholic beverages	Before	0 (0-4)	0 (0-5)	2 (0-5)	0 (0-4)
	During	(0-4)	0 (0.4)	0 (0-6)	0 (0-4)
	P value	0.801	0.257	0.853	0.239
Fruit	Before	35 (16-58)	33 (16-51)	40 (17-61)	34 (19-50)
	During	35 (16-54)	38 (16-53)	36 (16-38)	33 (18-50)
	P value	0.059	0.472	**0.019**	0.134
Vegetables	Before	52 (28-79)	51 (29-68)	61 (37-88)	50 (24-73)
	During	53 (29-91)	50 (32-69)	62 (36-86)	49 (24-73)
	P value	**0.003**	**0.011**	**0.029**	0.813

Supplementary Table S3. Correlation between change in headache frequency and change in lifestyle habit scores during social distancing.

	Change in frequency		Change in intensity	
	Rho	P value	Rho	P value
MET, vigorous activity	-0.032	0.612	+0.116	0.062
MET, moderate activity	-0.050	0.421	+0.001	0.984
MET, walking	-0.040	0.523	-0.042	0.497
MET, total	-0.042	0.497	+0.020	0.747
Protein-rich foods	-0.031	0.613	+0.078	0.206
Carbohydrate-rich foods	+0.032	0.605	+0.016	0.801
Dairies	+0.108	0.082	+0.046	0.461
Eggs	-0.057	0.355	+0.023	0.712
Sweets/cakes/chocolate	-0.106	0.087	+0.042	0.496
Sauces	-0.027	0.661	+0.098	0.113
Tea/coffee	+0.077	0.214	+0.120	0.053
Alcohol-free beverages	-0.011	0.860	+0.105	0.090
Alcoholic beverages	-0.049	0.430	-0.021	0.734
Fruit	+0.115	0.063	+0.051	0.416
Vegetables	-0.043	0.485	+0.118	0.057
Insomnia Severity Index score	+0.208	**0.001**	+0.160	**0.010**

Supplementary Figure S1. Box plots of changes in median METs (A), median monthly portions of carbohydrate-rich foods (B) and sweets.

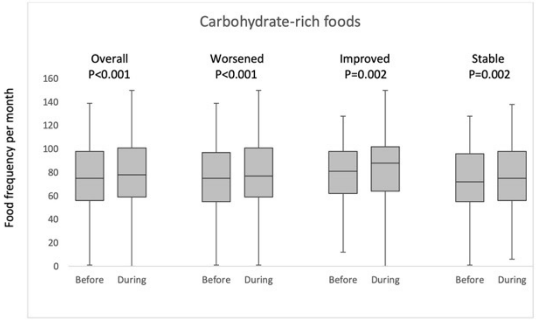

A

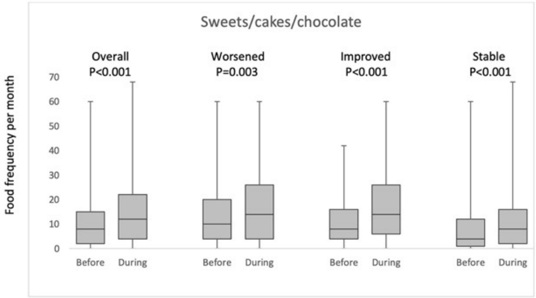

B
